# The Protective Effects of 5-Methoxytryptamine-α-lipoic Acid on Ionizing Radiation-Induced Hematopoietic Injury

**DOI:** 10.3390/ijms17060935

**Published:** 2016-06-14

**Authors:** Deguan Li, Zhenyuan Tian, Weisheng Tang, Junling Zhang, Lu Lu, Zhaojin Sun, Zewei Zhou, Feiyue Fan

**Affiliations:** Institute of Radiation Medicine, Chinese Academy of Medical Science and Peking Union Medical Collage, Tianjin Key Laboratory of Radiation Medicine and Molecular Nuclear Medicine, Tianjin 300192, China; lideguan@irm-cams.ac.cn (D.L.); tianzhenyuan1027@126.com (Z.T.); twsemail@126.com (W.T.); zhangjunling@irm-cams.ac.cn (J.Z.); lulu@irm-cams.ac.cn (L.L.); s654134369@126.com (Z.S.)

**Keywords:** ionizing radiation, hematopoietic cells, radiation protection, reactive oxygen species (ROS), radioprotectors, 5-methoxytryptamine-α-lipoic acid

## Abstract

Antioxidants are prospective radioprotectors because of their ability to scavenge radiation-induced reactive oxygen species (ROS). The hematopoietic system is widely studied in radiation research because of its high radiosensitivity. In the present study, we describe the beneficial effects of 5-methoxytryptamine-α-lipoic acid (MLA), which was synthesized from melatonin and α-lipoic acid, against radiation-induced hematopoietic injury. MLA administration significantly enhanced the survival rate of mice after 7.2 Gy total body irradiation. The results showed that MLA not only markedly increased the numbers and clonogenic potential of hematopoietic cells but also decreased DNA damage, as determined by flow cytometric analysis of histone H2AX phosphorylation. In addition, MLA decreased the levels of ROS in hematopoietic cells by inhibiting NOX4 expression. These data demonstrate that MLA prevents radiation-induced hematopoietic syndrome by increasing the number and function of and by inhibiting DNA damage and ROS production in hematopoietic cells. These data suggest MLA is beneficial for the protection of radiation injuries.

## 1. Introduction

With the wide use of ionizing radiation (IR) for therapeutic applications, the risk of radiation injury has increased [1,2]. IR may cause dysfunction in multiple organ systems, including the hematopoietic system, the gastrointestinal tract, the cerebrovascular systems, and even death. It is important to protect humans from IR-induced genotoxicity or lethality. Several studies have focused on the scavenging activities of antioxidants for IR-induced reactive oxygen species (ROS) [3,4,5] and have suggested that antioxidants may confer protection against IR injury.

**Figure 1:**
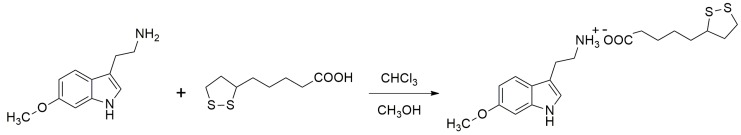
Synthesis and structure of 5-methoxytryptamine-α-lipoic acid (MLA).

Melatonin (*N*-acetyl-5-methoxytryptamine, MLT) is an indole hormone that is synthesized from the amino acid tryptophan and secreted by the pineal gland in mammals and humans [6,7]. In addition, α-lipoic acid (LA) is an endogenously produced co-factor of an enzyme that plays an important role in α-ketoacid dehydrogenase reactions [8,9]. MLT and LA are both effective endogenous ROS scavengers that have been shown to provide protection against IR injury [10,11,12,13]. In this study, LA was used as the substrate to synthesize a new compound by inserting the MLT amino acid functional groups related to anti-radical, anti-radiation, and immune activity. Using this procedure, we successfully synthesized a new antioxidant compound from MLT and LA, which we named 5-methoxytryptamine-α-lipoic acid (MLA). The synthesis and structure of MLA are shown in Figure 1.

In this study, we first confirmed that MLA could increase the survival rate of mice exposed to 7.2 Gy total body irradiation (TBI). Second, we examined the protective effects of MLA on the hematopoietic system in mice exposed to 6 Gy TBI, which could result in virtually no lethality for up to 30 days post-irradiation [14]. Finally, we investigated the mechanism by which MLA provides protection against IR injury. We demonstrated that MLA might mitigate IR-induced injury to the hematopoietic system by decreasing ROS levels and inhibiting NOX4 expression.

## 2. Results

### 2.1. Thirty-Day Survival after Exposure to 7.2 Gy Total Body Irradiation (TBI)

**Figure 2:**
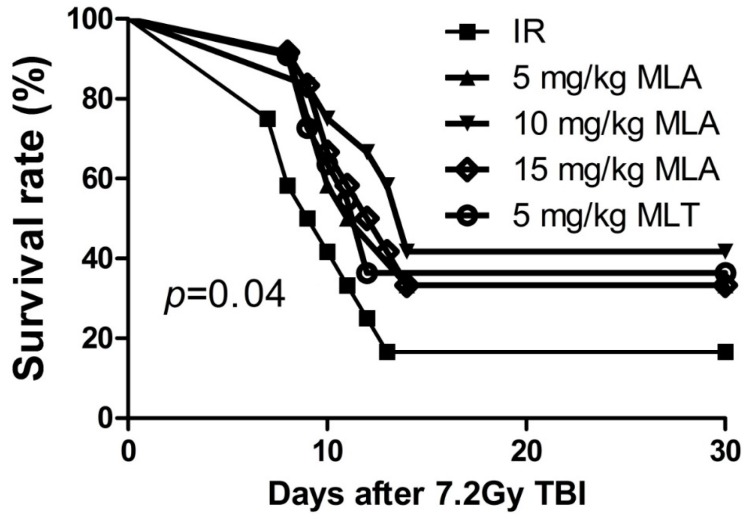
Effects of MLA on the survival of mice exposed to TBI (7.2 Gy). Before the mice (*n* = 12) were exposed to 7.2 Gy TBI, they were treated with different doses of MLA and MLT as described in the Materials and Methods section. The data are expressed as the percentage of surviving mice and were analyzed using the log-rank (Mantel–Cox) test. The *p* value shows the difference of 10 mg/kg MLT compared to the IR group.

To determine the protective effects of MLA on TBI-induced lethality in mice, we first observed the survival rates of mice after exposure to a lethal dose (7.2 Gy) of TBI. As shown in Figure 2, TBI resulted in mortality in 83% of the vehicle-treated mice by day 30 after irradiation. In the irradiated mice treated with 5 mg/kg MLA, 10 mg/kg MLA, 15 mg/kg MLA, or 5 mg/kg MLT, mortality was observed in 67%, 58%, 67% and 67% of the mice at day 30. The 30-day survival rate after TBI was 42% for mice treated with 10 mg MLA, which was significantly different from the vehicle-treated irradiated mice (*p* < 0.05). These results suggest that 10 mg/kg MLA effectively mitigates the TBI-induced lethality in mice.

### 2.2. 5-Methoxytryptamine-α-lipoic Acid (MLA) Elevated Peripheral Blood Cell Counts after Exposure to 6 Gy TBI

**Figure 3:**
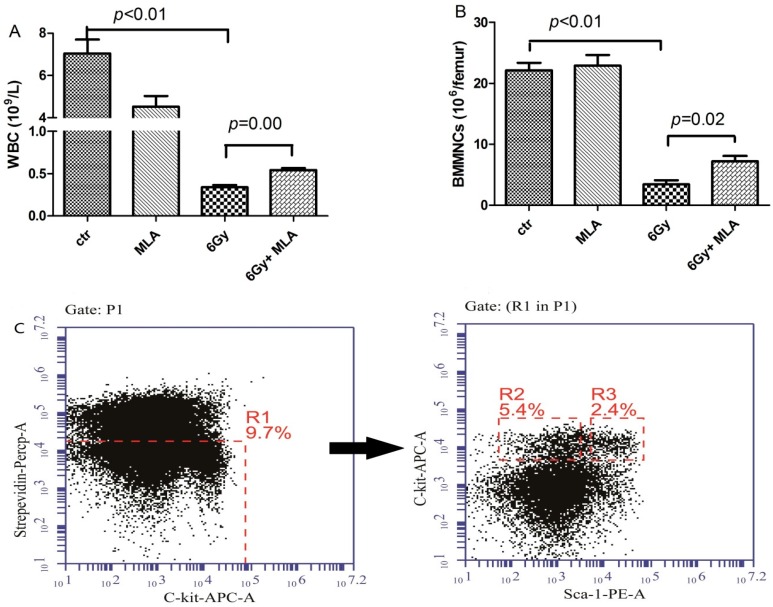

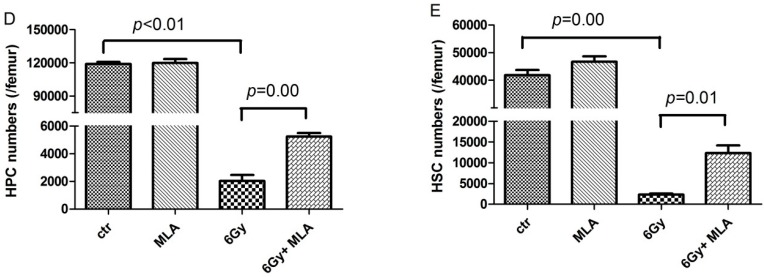
The effects of MLA on the numbers of BMMBNCs, HPCs, and HSCs. (**A**) Number of WBCs; (**B**) number of BMMNCs; (**C**) a representative flow cytometry graph: The BMMNCs were firstly gated by Strepevidin–Percp and C-kit-APC, and the HPC (lin^−^ c-kit^+^ Sca-1^−^,R2) and HSC (lin^−^ c-kit^+^ Sca-1^+^,R3) cells were then analyzed in the lineage negative cells (Strepevidin^−^, R1); (**D**) number of HPCs; E) number of HSCs. Before the mice were exposed to 6 Gy TBI, they were treated with either vehicle or MLA (10 mg/kg) each day three times. Sham-irradiated control mice and MLA-treated mice were also included. WBCs and BMMNCs were collected and counted after the mice were euthanized nine days after exposure to 6 Gy TBI. The numbers of HPCs (**D**) and HSCs (**E**) were calculated using the following formula: BMMNCs per femur multiply the HPC or HSC ratio detected by flow cytometry. Data are expressed as the mean ± SEM (*n* = 5 for each group). The *p* value shows the difference compared with the IR group.

To determine whether the reduced TBI-induced lethality from MLA was due to increased hematopoiesis, we analyzed peripheral blood cell counts nine days after the mice were exposed to a sub-lethal dose (6 Gy) of TBI (Figure 3A). Compared with the control mice (7.0 ± 1.5) × 10^9^ L^−1^, the number of white blood cells (WBC) in the IR group (0.3 ± 0.1) × 10^9^ L^−1^ was significantly decreased (*p* < 0.01). The treatment with MLA slightly elevated the WBC count (0.5 ± 0.1) × 10^9^ L^−1^, *p* < 0.01.

In addition, the numbers of bone marrow mononucleated cells (BMMNCs), hematopoietic progenitor cells (HPCs), and hematopoietic stem cells (HSCs) from each femur were also determined (Figure 3B,D,E). The numbers of BMMNCs (3.4 ± 1.2) × 10^6^, HPCs (2.0 ± 0.7) × 10^3^, and HSCs (2.3 ± 0.4) × 10^3^ femur^−1^ were significantly reduced in the vehicle-treated irradiated mice compared with the non-irradiated control group (22.1 ± 2.5) × 10^6^ BMMNCs, (11.9 ± 0.3) × 10^4^ HPCs, and (41.9 ± 2.6) × 10^3^ HSCs femur^−1^, *p* < 0.05. The numbers of BMMNCs (7.2 ± 1.9) × 10^6^, HPCs (11.4 ± 0.3) × 10^3^, and HSCs (12.4 ± 3.2) × 10^3^ femur^−1^ were increased in the MLA-treated irradiated mice compared with the vehicle-treated mice, indicating that the MLA treatment promoted hematopoiesis after TBI.

### 2.3. The Effect of MLA on the Colony Form Unit Granulocyte and Monocyte (CFU-GM) Frequency and the Number of CFU-S after Exposure to 6 Gy TBI

**Figure 4:**
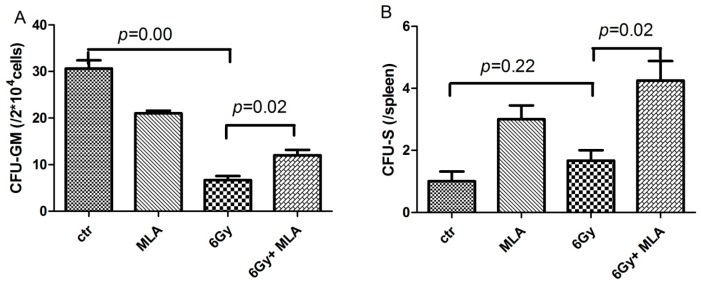
Effects of MLA on the CFU-GM frequency and the CFU-S number. (**A**) The CFU-GM frequency of BMMNCs. Before the mice were exposed to 6 Gy TBI, they were treated with either vehicle or MLA (10 mg/kg) each day three times. Sham-irradiated control mice and MLA-treated mice were also included. BMMNCs were collected and counted after the mice were euthanized nine days after exposure to 6 Gy TBI. The cells were cultured in MethoCult GF M3534 methylcellulose medium for CFU-GM analysis; (**B**) Number of CFU-S. The number of CFU-S per spleen was counted after the spleens were fixed in Bouin’s solution for 24 h. Results are expressed as the mean ± SEM (*n* = 5 for each group). The *p* value shows the difference compared with the IR group.

To determine whether MLA increased hematopoiesis after TBI exposure by stimulating hematopoietic progenitor cells, we examined the effect of MLA on the CFU-GM frequency and the number of CFU-S. As shown in Figure 4A, the CFU-GM frequency (6.7 ± 1.5) of CFU-GM in 2 × 10^4^ BM cells from vehicle-treated irradiated mice was significantly lower compared with the control mice (30.7 ± 3.0); *p* < 0.01. The reduced CFU-GM frequency was slightly attenuated by treatment with MLA (12.0 ± 2.0). The number of CFU-S in the 6 Gy TBI + MLA group (4.3 ± 1.2 spleen^−1^) was significantly higher than that in the 6 Gy TBI group (1.7 ± 0.5 spleen^−1^, *p* < 0.05, Figure 4B).

### 2.4. The Effect of MLA on BM Mononuclear Cell (BMMNC) Apoptosis after Exposure to 6 Gy TBI

**Figure 5:**
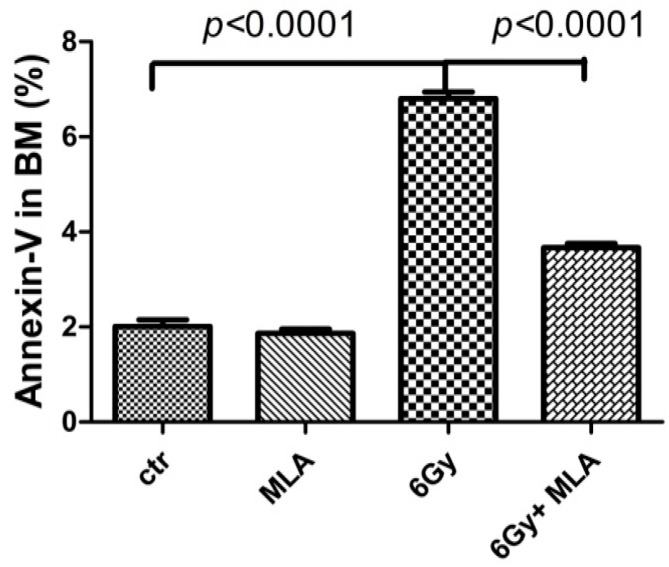
Effects of MLA on apoptosis in BMMNCs. Before the mice were exposed to 6 Gy TBI, they were treated with either vehicle or MLA (10 mg/kg) each day three times. Sham-irradiated control mice and MLA-treated mice were also included. BMMNCs were collected on day 9 and stained with Annexin V-FITC to evaluate apoptosis by flow cytometry. Results are expressed as the mean ± SEM. The *p* value shows the difference compared with the IR group.

To determine whether MLA elevated hematopoiesis by decreasing apoptosis, BM cells were stained with Annexin V-FITC and PI, and analyzed by flow cytometry after mice were exposed to 6 Gy TBI for nine days. As shown in Figure 5, the apoptotic BM cells rate (6.8% ± 0.3%) in the irradiated mice was significantly elevated by IR (*p* < 0.01). The increase in apoptosis was attenuated by MLA (3.7% ± 0.2%, *p* < 0.01). These results suggested that MLA could protect hematopoietic cells from radiation-induced injury by inhibiting apoptosis [15].

### 2.5. The Effects of MLA on Hematopoietic DNA Injury after Exposure to 6 Gy TBI

**Figure 6:**
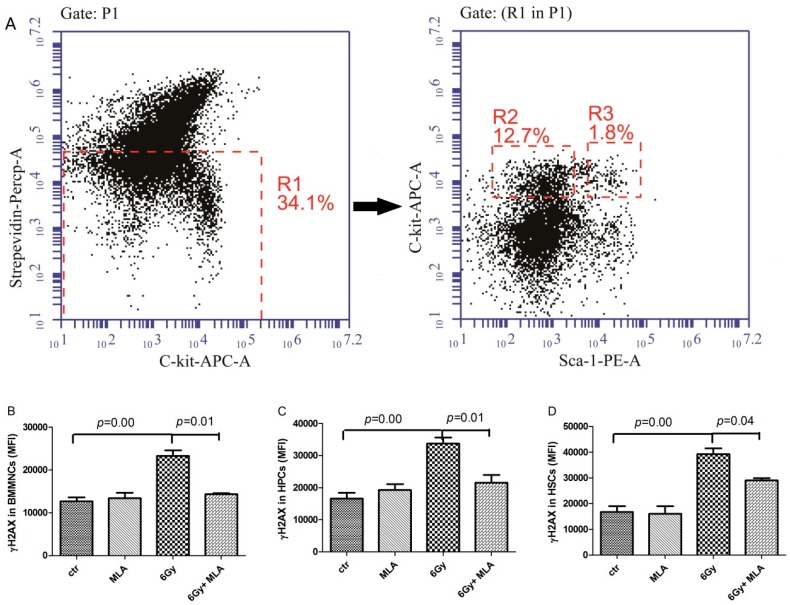
Effects of MLA on the H2AX phosphorylation. (**A**) A representative flow cytometry graph: The BMMNCs(P1) were firstly gated by Strepevidin–Percp and C-kit-APC, then the HPC (lin^−^ c-kit^+^ Sca-1^−^, R2) and HSC (lin^−^ c-kit^+^ Sca-1^+^, R3) cells were gated in the lineage negative cells (Strepevidin^−^, R1), and the H2AX phosphorylation in these cells were further analyzed; (**B**) H2AX phosphorylation in BMMNCs; (**C**) H2AX phosphorylation in HPCs; (**D**) H2AX phosphorylation in HSCs. Before the mice were exposed to 6 Gy TBI, they were treated with either vehicle or MLA (10 mg/kg) each day three times. Sham-irradiated control mice and MLA-treated mice were also included. BMMNCs were collected on day 9 and detected according to the Materials and Methods section. Results are expressed as the mean ± SEM. The *p* value shows the difference compared with the IR group.

We examined whether MLA treatment could reduce TBI-induced DNA damage by flow cytometry analysis of histone H2AX phosphorylation, which has been widely used as a marker for DNA double-strand breaks (DSBs). As shown in Figure 6B–D, there was an increase in H2AX phosphorylation in BMMNCs (23.3 ± 2.9) × 10^3^, HPCs (33.4 ± 3.8) × 10^3^, and HSCs (39.3 ± 4.5) × 10^3^ from the IR group compared with the control group (12.6 ± 2.1) × 10^3^, (16.6 ± 4.0) × 10^3^, (16.7 ± 5.1) × 10^3^, respectively. MLA treatment decreased H2AX phosphorylation in all three hematopoietic cells (BMMNCs: (14.3 ± 0.3) × 10^3^, HPCs: (21.5 ± 4.2) × 10^3^, and HSCs: (29.1 ± 1.3) × 10^3^) compared with the irradiated mice (*p* < 0.05).

### 2.6. The Effects of MLA on Reactive Oxygen Species(ROS) Levels in Hematopoietic Cells after Exposure to 6 Gy TBI

**Figure 7:**
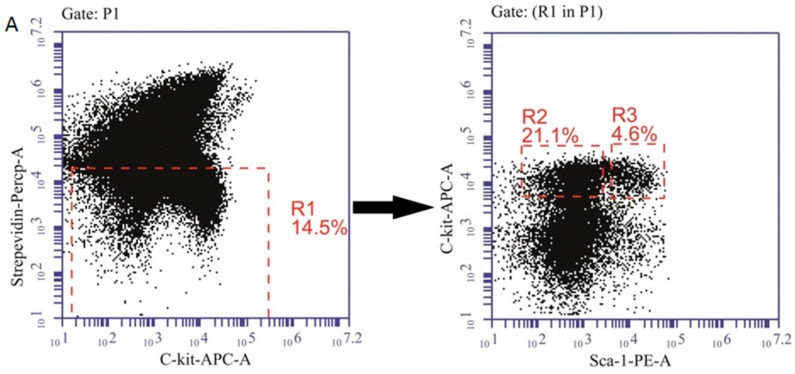

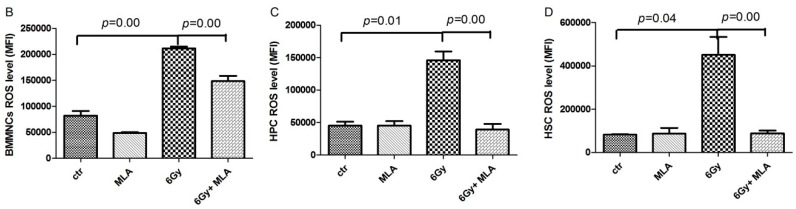
Effects of MLA on the levels of ROS. (**A**) A representative flow cytometry graph: The BMMNCs(P1) were firstly gated by Strepevidin–Percp and C-kit-APC, then the HPC (lin^−^ c-kit^+^ Sca-1^−^, R2) and HSC (lin^−^ c-kit^+^ Sca-1^+^, R3) cells were gated in the lineage negative cells (Strepevidin^−^, R1), and the ROS levels in these cells were further analyzed; (**B**) ROS levels in BMMNCs; (**C**) ROS levels in HPCs; (**D**) ROS levels in HSCs. Before the mice were exposed to 6 Gy TBI, they were treated with either vehicle or MLA (10 mg/kg) each day three times. Sham-irradiated control mice and MLA-treated mice were also included. BMMNCs were collected and detected according to the Methods. The results are expressed as the mean ± SEM. The *p* value shows the difference compared with the IR group.

To identify the mechanism underlying the effect of MLA, the ROS levels were detected in BMMNCs, HPCs, and HSCs. As shown in Figure 7B–D, there was an increase in ROS levels in BMMNCs (21.2 ± 0.6) × 10^4^, HPCs (14.6 ± 2.3) × 10^4^, and HSCs (45.1 ± 14.3) × 10^4^ in the IR group compared with the control group (8.2 ± 1.8) × 10^4^, (4.5 ± 0.8) × 10^4^, (8.3 ± 0.1) × 10^4^, respectively. The MLA treatment decreased the ROS levels in all three hematopoietic cells (BMMNCs: (14.9 ± 2.0) × 10^4^, HPCs: (13.9 ± 1.7) × 10^4^, and HSCs: (8.8 ± 2.5) × 10^4^) after exposure to 6 Gy TBI (*p* < 0.01).

### 2.7. The Effects of MLA on the Expression of NOX4 in Hematopoietic Cells after Exposure to 6 Gy TBI

**Figure 8:**
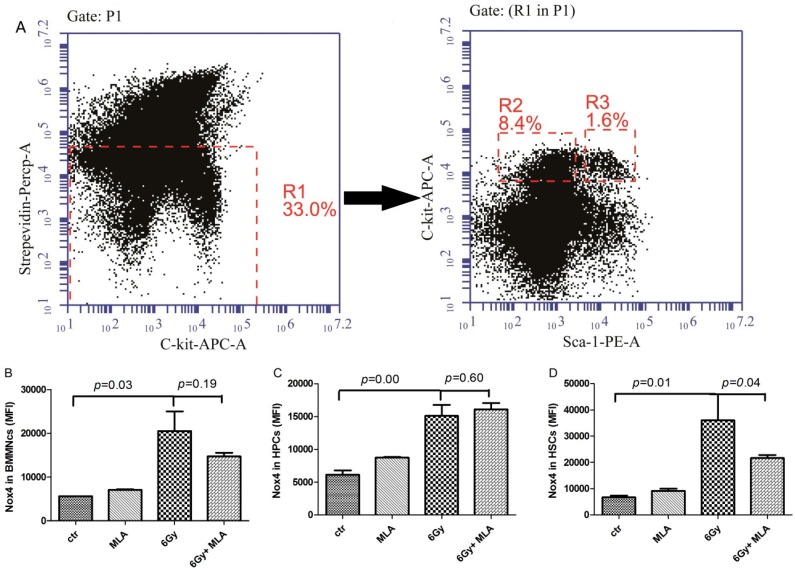
Effects of MLA on the expression of NOX4. (**A**) A representative flow cytometry graph: The BMMNCs(P1) were firstly gated by Strepevidin–Percp and C-kit-APC (R1), then the HPC (lin^−^ c-kit^+^ Sca-1^−^, R2) and HSC (lin^−^ c-kit^+^ Sca-1^+^, R3) cells were gated in the lineage negative cells (Strepevidin^−^, R1), and the NOX4 expression in these cells were further analyzed; (**B**) NOX4 expression in BMMNCs; (**C**) NOX4 expression in HPCs; (**D**) NOX4 expression in HSCs. Before the mice were exposed to 6 Gy TBI, they were treated with either vehicle or MLA (10 mg/kg) each day three times. Sham-irradiated control mice and MLA-treated mice were also included. BMMNCs were collected and detected according to the Methods. Results are expressed as the mean ± SEM. The *p* value shows the difference compared with the IR group.

Our previous studies have shown that NOX4 plays an important role in ROS production in hematopoietic cells [16]. Therefore, we examined the effects of MLA on the expression of NOX4. As shown in Figure 8B–D, an increase in NOX4 expression was detected in BMMNCs (20.5 ± 7.7) × 10^3^, HPCs (15.1 ± 2.8) × 10^3^, and HSCs (3.63 ± 1.4) × 10^3^ in the IR group compared with the control group (5.6 ± 0.0) × 10^3^; (6.1 ± 1.3) × 10^3^; (6.7 ± 1.3) × 10^3^; respectively. MLA treatment decreased the expression of NOX4 only in HSCs (21.7 ± 2.0) × 10^3^ after exposure to 6 Gy TBI (*p* < 0.05).

## 3. Discussion

Ionizing radiation can induce various pathophysiological alterations, including direct DNA damage and indirect oxidative stress, which contribute to irradiation-induced injury in multiple tissues [17]. The development of an effective treatment for radiation protection and therapy remains an important area of research [18]. Melatonin, which was discovered approximately 40 years ago, has been studied widely and is used as a direct free radical scavenger and an indirect antioxidant. Previous studies have demonstrated that MLT can ameliorate radiation injury in different tissues [19,20]. Sharma showed that 30 mg/kg MLT increased immunity in squirrels by protecting the hematopoietic system and lymphoid organs after exposure to 2.06 Gy X-ray radiation [21]. Shirazi showed that 250 mg/kg MLT might reduce liver and peripheral blood radiation injury in rats [22,23]. In addition, MLT was used in the radiation leak from the Fukushima Daiichi Nuclear Power Plant to protect individuals in the fallout zone and workers in the Daiichi complex [24]. α-Lipoic acid (LA) is a natural product that was isolated in 1951 from beef liver and has been shown to quench ROS in both lipid and aqueous phases, chelate transition metals, and prevent membrane lipid peroxidation and protein damage [25,26]. Ramachandran showed that 20–100 mg LA could prevent BM cell chromosomal damages and the formation of micronuclei, but could not elevate mice survival rate [27]. In the present study, we synthesized a new antioxidant compound (MLA) and explored its ability on the 30-day survival rate of irradiated mice. Up to 200 mg/kg MLA showed no toxic effects on the mice. Though there were no obvious differences between 5 mg/kg MLA and MLT, the higher molecular weight of MLA might be the reason. 10 mg/kg MLA could slightly elevate the mice’s 30-day survival rate, the effective doses were lower than MLT and LA published previously [20,21,27,28].

In the present study, MLA improved the survival rates of mice after exposure to a lethal dose of radiation, confirming that MLA provided protection against radiation injury [28,29]. Hematopoietic injury is one of the most important side effects of radiation syndrome [30,31]; therefore, we investigated the effects of MLA on the hematopoietic system using an established mouse model [14]. Chlorophyllin, lycopene, vitamin E, and other radioprotectors all show protective effects on the peripheral blood [32,33,34,35,36]. We found that MLA increased both the numbers of BMMNCs, HPCs, and HSCs, and the colony-forming ability of BM cells, which suggested that MLA not only protected the cells against IR injury but also improved their function.

It is well-known that most IR injuries result from ROS generated in response to IR exposure, and cellular ROS levels are commonly elevated by IR. Antioxidants are known to scavenge free radicals and are therefore considered good candidates for radioprotectors [37]. In the present study, ROS levels in BMMNCs, HPCs, and HSCs were detected by flow cytometry. The results indicated that MLA could effectively decrease the ROS levels in BMMNCs, HPCs, and HSCs, demonstrating that MLA is a potent antioxidant.

ROS induced by IR can disrupt the structure and function of DNA, lipids, and proteins, which leads to metabolic and functional alterations and ultimately to cell apoptosis, senescence or necrosis [18,38]. In this study, the effect of MLA on BM cell apoptosis was evaluated. The results showed that MLA could significantly decrease the rate of IR-induced BM cell apoptosis, which was consistent with the observed changes in cell counts. Among the various macromolecules, DNA is considered to be the most critical molecular target for IR-induced cell injury and death. Previous studies have demonstrated that the inhibition of ROS can decrease IR-induced DNA damages such as DNA double-strand breaks (DSB) and single-strand breaks. The DSB will result in the phosphorylation of H2AX histones around each DSB to form γH2AX foci and can be detected by a Ser139-specific antibody. H2AX phosphorylation has been used for quantifying DSBs in many studies [39,40]. In the present study, we analyzed the H2AX phosphorylation in hematopoietic cells. The results indicated that MLA could mitigate the DNA injury induced by IR.

The mechanisms involved in the efficacy of MLA treatment initiated after radiation exposure remain to be elucidated, particularly in the context of ROS signaling. Previous studies have shown that NOX4 plays an important role in ROS production in HSC cells [16,41,42]. Therefore, the expression of NOX4 in hematopoietic cells was evaluated by flow cytometry. The results showed that MLA can effectively decrease NOX4 expression in HSCs, which was consistent with previous results from our laboratory [43].

Our studies demonstrate the utility of MLA for protection against radiation-induced injury. We showed that MLA may protect mice against IR injury by decreasing ROS levels through the inhibition of NOX4 expression. This research suggests that MLA may be a new therapeutic drug for radiation protection.

## 4. Materials and Methods

### 4.1. Ethics Statement

Animals were housed in the certified animal facility (Specific Pathogen Free level) at Institute of Radiation Medicine (IRM), Chinese Academy of Medical Sciences (CAMS). All procedures involving animals were reviewed and approved by the Animal Care and Use Committee (ACUC) of IRM (Permit Number 1526, 7 April 2015) written informed consent was obtained from all participants. For the survival study, irradiated mice were monitored twice a day for clinical signs as described in the ACUC-IRM policy to categorize animals as morbid or moribund. When an animal met the definitive criteria for moribundity (abdominal breathing, inability to stand, or inability to right itself within 5 s when placed gently on its side), it was humanely euthanized at an early endpoint using 100% CO_2_ inhalation followed by cervical dislocation, in accordance with the American Veterinary Medical Association (AVMA) Guidelines for the Euthanasia of Animals.

### 4.2. Reagents

The anti-mouse Ly-6A/EA (Sca-1)-PE/Cy7, CD117 (c-kit), APC, biotin-conjugated CD5, CD4, CD8, CD45R/B220, Ly6G/Gr-1, CD11b, Ter-119, and APC/CY7-conjugated streptavidin antibodies, and an Annexin V-FITC apoptosis kit were purchased from eBioscience (San Diego, CA, USA). The 2′,7′-dichlorodihydrofluorescein diacetate (DCFDA), α-lipoic acid, and 5-methoxytryptamine were purchased from Sigma-Aldrich (St. Louis, MO, USA). The RPMI 1640 medium was purchased from Gibco (Grand Island, NY, USA). The BD Cytofix/Cytoperm buffer was purchased from BD Biosciences (San Diego, CA, USA). Methylcellulose M3534 was purchased from Stem Cell (Vancouer, BC, Canada). Fetal calf serum was purchased from Biological Industries (Kibbutz, Israel). The rabbit anti-γH2AX antibody was obtained from Cell Signaling Technology (Danvers, MA, USA), the rabbit anti-NOX4 antibody from Proteintech (Wuhan, China) and the FITC-conjugated goat anti-rabbit antibodies from Abcam (Cambridge, MA, USA).

The 5-methoxytryptamine-α-lipoic acid (MLA) was synthetized using α-lipoic acid and 5-methoxytryptamine at the drug department of the Institute of Radiation Medicine, CAMS. Melatonin (MLT) was purchased from Tokyo Chemical Industry Co., Ltd. (Shanghai, China). MLA and MLT were dissolved in a 5% carboxymethyl cellulose (CMC) solution.

### 4.3. Animals

Male C57BL/6 mice weighing 20–22 g were purchased from Beijing HFK Bioscience Co., Ltd. (Beijing, China) and housed in the certified animal facility at the Institute of Radiation Medicine, CAMS.

### 4.4. Irradiation and Treatment

Irradiation was performed using a ^137^Cs source housed in an Exposure Instrument Gammacell-40 (Atomic Energy of Canada Lim, Chalk River, ON, Canada) at a dose-rate of 1.0 Gy per minute. Sham-irradiated mice were treated similarly to the irradiated mice but without exposure to IR. After irradiation, the mice were returned to the animal facility for daily observation and treatment as described below. The mice were exposed to 7.2 Gy TBI in the survival experiments [44] and 6.0 Gy TBI in the remaining experiments [14].

The 72 mice in the survival experiments were randomly assigned to 6 treatment groups: control, vehicle + 7.2 Gy TBI, 5 mg/kg MLA + 7.2 Gy TBI, 10 mg/kg MLA + 7.2 Gy TBI, 15 mg/kg MLA + 7.2 Gy TBI, and 5 mg/kg MLT + 7.2 Gy TBI. For the MLA treatment, the mice were administered 0.2 mL of solution by gavage 3 times over the 3 days prior to irradiation. For the MLT treatment, the mice were administered 0.2 mL of solution by gavage 3 times over the 3 days prior to irradiation. The control and 7.2 Gy TBI groups were treated with vehicle similarly to the procedure described for the MLA and MLT treatments. All the mice in irradiated groups were irradiated 1 h after the last treatment.

The 20 mice in the remaining experiments were randomly assigned to 4 treatment groups: control, 10 mg/kg MLA + 6.0 Gy TBI, vehicle + 6.0 Gy TBI, and 10 mg/kg MLA + 6.0 Gy TBI. The mice were treated as described above and were killed 9 days after exposure to irradiation.

### 4.5. Peripheral Blood Cell and BMMNC Counts

Blood samples were obtained from the orbital sinus after mice were exposed to 6 Gy TBI for 10 days. The bone marrow (BM) cells were flushed from mouse femurs with PBS after the mice were euthanized. The numbers of various blood cell types and BMMNCs were counted using a MEK-7222k hemocytometer (NIHON KOHDEN Corp., Tokyo, Japan) and expressed as 10^9^ L^−1^ and 10^6^ femur^−1^, respectively.

### 4.6. Detection of Hematopoietic Progenitor Cells (HPCs) and Hematopoietic Stem Cells (HSCs)

In brief, BM cells were incubated with biotin-conjugated lineage antibodies specific for murine CD5, Ter119, CD11b, CD45R/B220, and Gr-1 and stained with streptavidin-APC-Cy7, Sca1-PE-Cy7, and c-kit-APC. The HPC (lin^−^ c-kit^+^ Sca-1^−^) and HSC (lin^−^ c-kit^+^ Sca-1^+^, LSK) numbers were calculated using the following equation: percentage × BMMNCs/femur [15].

### 4.7. Colony-Forming Cell (CFC) Assay

The CFC assay was performed by culturing BMMNCs in MethoCult GF M3534 methylcellulose medium (Stem Cell Technologies, Vancouver, BC, Canada). Colony-forming unit granulocyte-macrophage (CFU-GM) colonies were counted on day 7 post-irradiation using a microscope, according to the manufacturer’s protocol and expressed by per 2 × 10^4^ cells [45].

### 4.8. Endogenous Spleen Colony-Forming Units

Spleens were removed from the mice and fixed in Bouin′s solution (trinitrophenol and methanal) for 24 h. Macroscopic spleen colony-forming units (CFU-S; visible to the naked eye) were scored for each spleen [46,47].

### 4.9. Intracellular ROS Analysis

After the BM cells were stained with the LSK antibodies as described above, the cells were incubated with 10 μM DCFDA for 20 min at 37 °C. The intracellular ROS levels in hematopoietic cells were analyzed by measuring the mean fluorescence intensity (MFI) of DCF by flow cytometry. For each sample, a minimum of 100,000 Lin-cells was acquired [48].

### 4.10. Analysis of H2AX Phosphorylation and NOX4 Expression

After the BM cells were stained with the LSK antibodies as described above, the cells were fixed and permeabilized with BD Cytofix/Cytoperm buffer according to the manufacturer’s protocol. The cells were then stained with antibodies against H2AX phosphorylation or NOX4 and FITC-conjugated secondary antibodies. The H2AX phosphorylation and expression of NOX4 in the hematopoietic cells was determined by analyzing the MFI of FITC by flow cytometry [43].
